# Spheroid formation of human thyroid cancer cells under simulated microgravity: a possible role of CTGF and CAV1

**DOI:** 10.1186/1478-811X-12-32

**Published:** 2014-05-10

**Authors:** Elisabeth Warnke, Jessica Pietsch, Markus Wehland, Johann Bauer, Manfred Infanger, Mark Görög, Ruth Hemmersbach, Markus Braun, Xiao Ma, Jayashree Sahana, Daniela Grimm

**Affiliations:** 1 Clinic for Plastic, Aesthetic and Hand Surgery, Otto-von-Guericke-University Magdeburg, Magdeburg, Germany; 2 Max-Planck Institute of Biochemistry, Martinsried, Germany; 3 DLR, German Aerospace Center, Institute of Aerospace Medicine, Cologne, Germany; 4 Institute for Molecular Physiology and Biotechnology of Plants (IMBIO), University of Bonn, Gravitational Biology Group, Kirschallee 1, 53115, Bonn, Germany; 5 Institute of Biomedicine, Pharmacology, Aarhus University, Wilhelm Meyers Allé 4, DK-8000 Aarhus C, Denmark

**Keywords:** Adherent growth, Three-dimensional growth, Cytokine, Microgravity, MCTS, RPM, 2D clinostat

## Abstract

**Background:**

Multicellular tumor spheroids (MCTS) formed scaffold-free under microgravity are of high interest for research and medicine. Their formation mechanism can be studied in space in real microgravity or on Earth using ground-based facilities (GBF), which simulate microgravity. On Earth, these experiments are more cost-efficient and easily performable. However, each GBF might exert device-specific and altered superimposingly gravity-dependent effects on the cells.

**Results:**

FTC-133 human thyroid cancer cells were cultivated on a 2D clinostat (CN) and a random positioning machine (RPM) and compared with corresponding 1 *g* control cells. Harvested cell samples were investigated by microscopy, quantitative realtime-PCR and Multi-Analyte Profiling. Spheroid formation and growth occurred during 72 h of cultivation on both devices. Cytokine secretion and gene activation patterns frequently altered in different ways, when the cells were cultured either on the RPM or the CN. A decreased expression of *CAV1* and *CTGF* in MCTS compared to adherent cells was observed after cultivation on both machines.

**Conclusion:**

The development of MCTS proceeds similarly on the RPM and the CN resembling the situation observed under real microgravity conditions, while no MCTS formation was observed at 1 *g* under identical experimental conditions. Simultaneously, changes in the regulation of *CTGF* and *CAV1* appeared in a comparable manner on both machines*.* A relationship between these molecules and MCTS formation is discussed.

## Background

During a recent flight on board the Shenzhou-8 spacecraft human follicular thyroid cancer cells (FTC-133) were exposed to real microgravity for 10 days [1,2]. The returned samples revealed that scaffold-free formation of multicellular tumor cell spheroids (MCTS) occurred while the cells had been exposed to real microgravity. The spheroids obtained after landing of the space ship return capsule showed a similar shape but larger diameters (5–10 mm) than those usually induced on Earth with the help of a random positioning machine (RPM) [1-4]. In contrast, 1 *g* controls kept under static conditions remained adherent and formed no spheroids. From these results we concluded that microgravity could be a major cause of transition from 2- to 3-dimensional cellular growth. However, involved molecules and signaling pathways responsible for this change of growth behavior remained unknown [5].

In order to understand and explain the effects of altered gravity on spheroid formation, we complemented our studies using two different ground-based facilities in order to simulate microgravity conditions – the 2D clinostat (CN) and the RPM. Both devices are cost-efficient and enable a sufficient number of experiments, which is rarely achieved under real microgravity conditions [6]. Each of these ground-based approaches prevents cell sedimentation, however, in a device-specific manner. On the clinostat, sedimentation is prevented by a fast and constant rotation of the samples around one horizontal axis, assuming that the sample does no longer perceive the gravity stimulus [7]. In contrast, the RPM consists of two independently rotating frames enabling a 3D rotation with random speed and random direction of the samples aiming to alter the influence of the gravity vector [8,9].

Considering the construction of both machines, we concluded that a permanent change of the direction of the gravity vector and thus prevention of sedimentation is a common capacity of both machines, while their particular modes of operations are rather different. Therefore, we aimed to analyze whether biological processes triggered by altered gravity may show identical or different results after exposure on these two kinds of devices.

In order to prove ground-based microgravity simulation approaches, we investigated human follicular thyroid cancer cells (FTC-133) cultivated either on the CN or the RPM in a parallel manner focusing on the formation of spheroids as well as on alterations of gene expression and protein secretion. We learned that spheroids are formed on both devices and concluded that caveolin-1 (CAV1) and connective tissue growth factor (CTGF) could be directly involved in the initiation of 3D cell growth.

## Results

### Spheroid formation on the RPM and the CN

**Figure 1:**
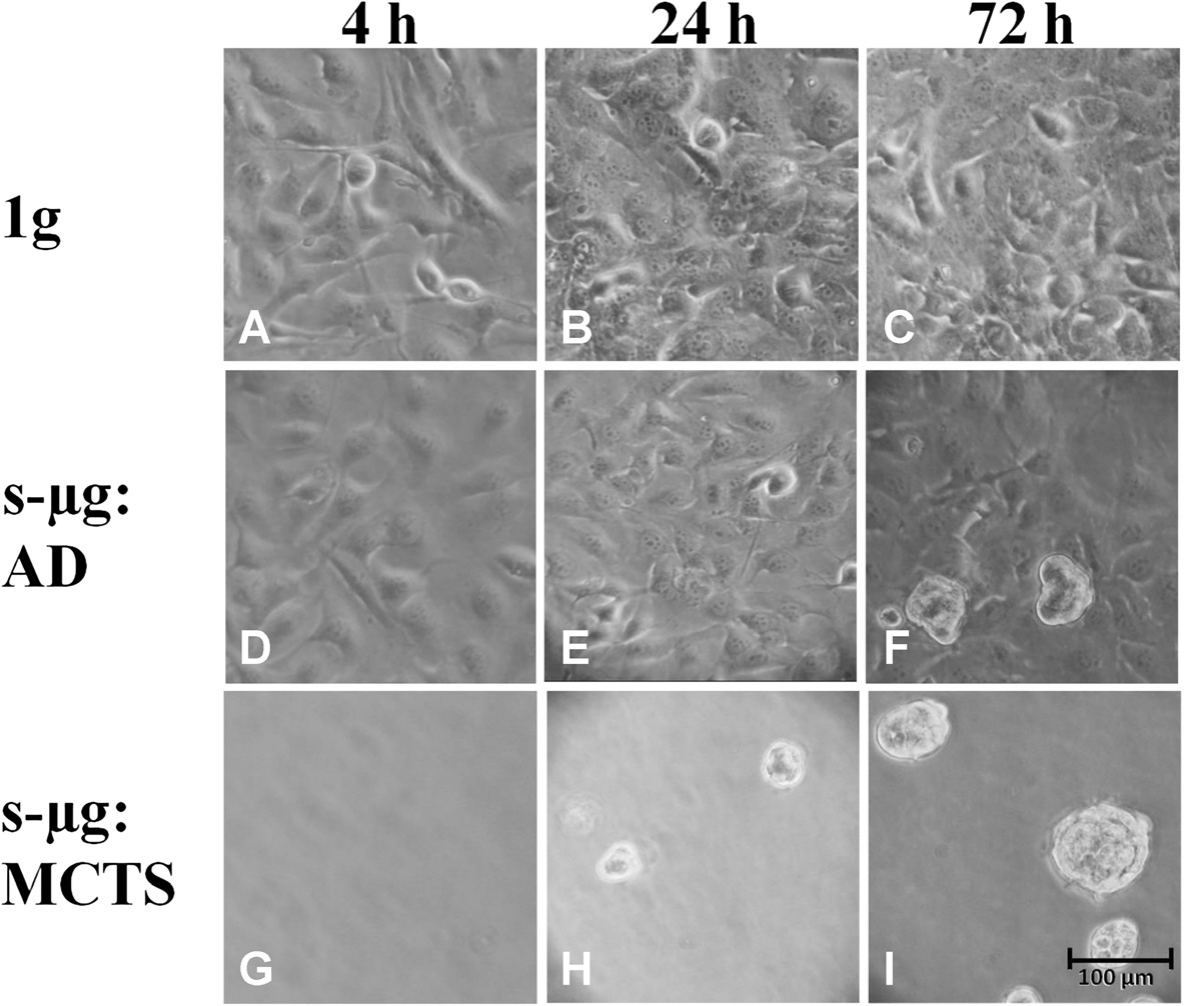
**Phase-contrast microscopy of FTC-133 after culturing on the CN or as corresponding 1g control.** The cells grew exclusively as adherent monolayers (AD) in 1 g controls **(A-C)**. On the CN, the FTC-133 cell population began to split in adherent cells **(E, F)** and MCTS cells **(H, I)** after 24 h clinorotation. 1 g control, adherent cells, and MCTS after 4 h **(A, D, G)**, 24 h **(B, E, H)**, and 72 h **(C, F, I)** of clinorotation.

Subconfluent monolayers of human follicular thyroid carcinoma cells (FTC-133) were cultivated either on the RPM or on the CN and in parallel to the 1 *g* controls located in the same incubator, respectively. On both devices spheroid formation progressed like shown in Figure 1 for cells harvested from the CN. While under static 1 *g* conditions (1 *g* controls), the cells remained adherent (Figure 1 A-C), two cell populations developed within the culture flasks mounted on each of the two machines, respectively (Figure 1 D-I). Of these populations, one continued to grow adherently (AD cells) (Figure 1 D-F), the other one detached from the bottom of the culture flask and assembled to MCTS (Figure 1 H-I). The separation of the two cell populations is delayed, as 4 h after exposure to the devices only adherent growth was observed in each sample (Figure 1 A, D, G). After approximately 24 h early spheroids became visible on each of both the devices in addition to the adherently growing cells (Figure 1 E, H). During the subsequent 48 h, spheroids became more numerous and larger, while adherently growing cells were still present (Figure 1 F, I). Spheroid size can be assumed to be around 100 μm on the clinostat, as shown in Figure 1, but also reaching up to 1 mm on the RPM as previously published by Pietsch *et al.*[4].

### Differential gene expression in FTC-133 cells after exposure to CN and RPM

After spheroid formation was observed on the RPM and the CN, we were interested to see whether similar alterations of mRNA expression occurred on both machines. Therefore, qPCR on several types of mRNA was performed, which had been recognized in former experiments on FTC-133 cells exposed to the RPM for 24 h to be important for MCTS formation [10]. The selected genes belong to several biological categories including cytoskeletal proteins, and factors of growth, apoptosis, angiogenesis and signal transduction (Table 1).

**Table 1:** Primers used for quantitative real-time PCR

***Gene***	***Primer name***	***Sequence***
*18S rRNA*	18S-F	GGAGCCTGCGGCTTAATTT
	18S-R	CAACTAAGAACGGCCATGCA
*CAV1*	CAV1-F	GTACGACGCGCACACCAA
	CAV1-R	TCCCTTCTGGTTCTGCAATCA
*CAV2*	CAV2-F	GATCCCCACCGGCTCAAC
	CAV2-R	CACCGGCTCTGCGATCA
*CD44*	CD44-F	ACCCTCCCCTCATTCACCAT
	CD44-R	GTTGTACTACTAGGAGTTGCCTGGATT
*CTGF*	CTGF-F	ACAAGGGCCTCTTCTGTGACTT
	CTGF-R	GGTACACCGTACCACCGAAGAT
*EGF*	EGF-F	TGCCAGCTGCACAAATACAGA
	EGF-R	TCTTACGGAATAGTGGTGGTCATC
*ERK1*	ERK1-F	ACCTGCGACCTTAAGATTTGTGA
	ERK1-R	AGCCACATACTCCGTCAGGAA
*IL8*	IL8-F	TGGCAGCCTTCCTGATTTCT
	IL8-R	GGGTGGAAAGGTTTGGAGTATG
*ITGB1*	ITGB1-F	GAAAACAGCGCATATCTGGAAATT
	ITGB1-R	CAGCCAATCAGTGATCCACAA
*NFKBP65*	NFKBP65-F	CGCTTCTTCACACACTGGATTC
	NFKBP65-R	ACTGCCGGGATGGCTTCT
*OPN*	OPN-F	CGAGGTGATAGTGTGGTTTAT GGA
	OPN-R	CGTCTGTAGCATCAGGGTACTG
*PRKCA*	PKCC-F	CATTCAACAGCTGGGCAAGTT
	PKCC-R	GTAGATGATGCCCTGATTGTGAAG
*TLN1*	TLN1-F	GATGGCTATTACTCAGTACAGACAACTGA
	TLN1-F	CATAGTAGACTCCTCATCTCCTTCCA
*VEGFA*	VEGFA-F	GCGCTGATAGACATCCATGAAC
	VEGFA-R	CTACCTCCACCATGCCAAGTG
*VEGFD*	VEGFD-F	TGCAGGAGGAAAATCCACTTG
	VEGFD-R	CTCGCAACGATCTTCGTCAA
*NGAL*	NGAL-F	AGGGAGTACTTCAAGATCACCCTCTA
	NGAL-R	AGAGATTTGGAGAAGCGGATGA
*MSN*	MSN-F	GAAATTTGTCATCAAGCCCATTG
	MSN-R	CCATGCAAGGCCAAGAT

All sequences are given in 5’-3’ direction.

**Figure 2:**
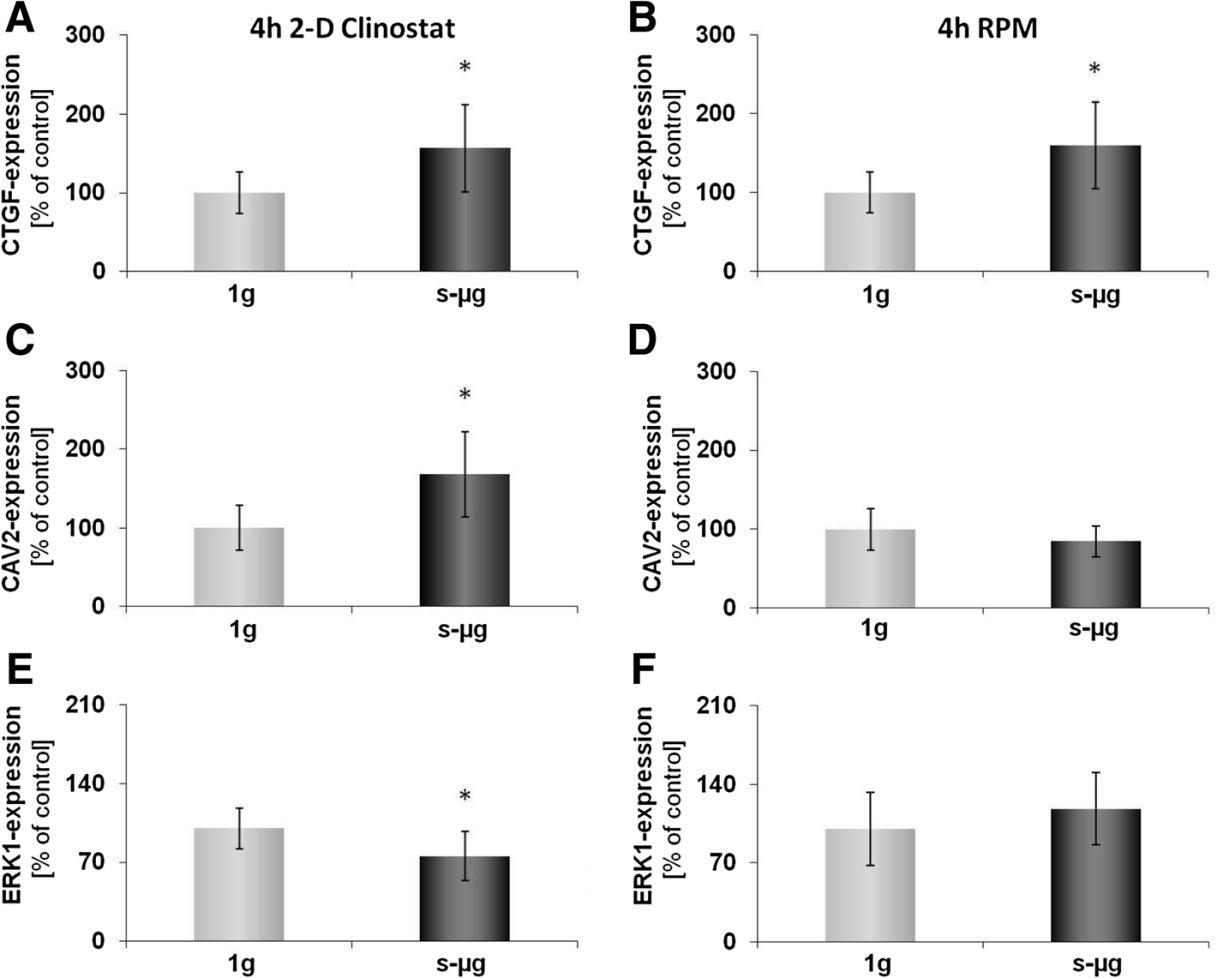
**Quantitative real-time PCR for the determination of alterations in gene-expression of selected genes after 4 h.** CTGF **(A, B)**, CAV2 **(C, D)** and ERK1 **(E, F)** gene expression was analyzed after 4 h exposure of the cells to 2-D Clinostat **(A, C, E)** or random positioning machine (RPM; **B**, **D**, **F**). CTGF **(A, B)** was upregulated on both machines while CAV2 **(C)** and ERK1 **(E)** were differentially expressed on the CN only. All results are shown as mean ± standard deviation (SD) of n = 10 independent samples, with significance indicated by *P < 0.05 vs. 1g.

As shown in Figure 1, after a 4 h-incubation on the RPM and on the CN the whole FTC-133 population grew adherently like in 1 *g-*control samples. Nevertheless, some mRNA changes were already found in cells harvested from either machine as compared to control cells. An up-regulation in the *CTGF* gene expression was observed in cell cultures on both devices. However, an up-regulation of *CAV2* and a down-regulation of *ERK1* gene expression were only evident after culturing on the CN (Figure 2).

**Figure 3:**
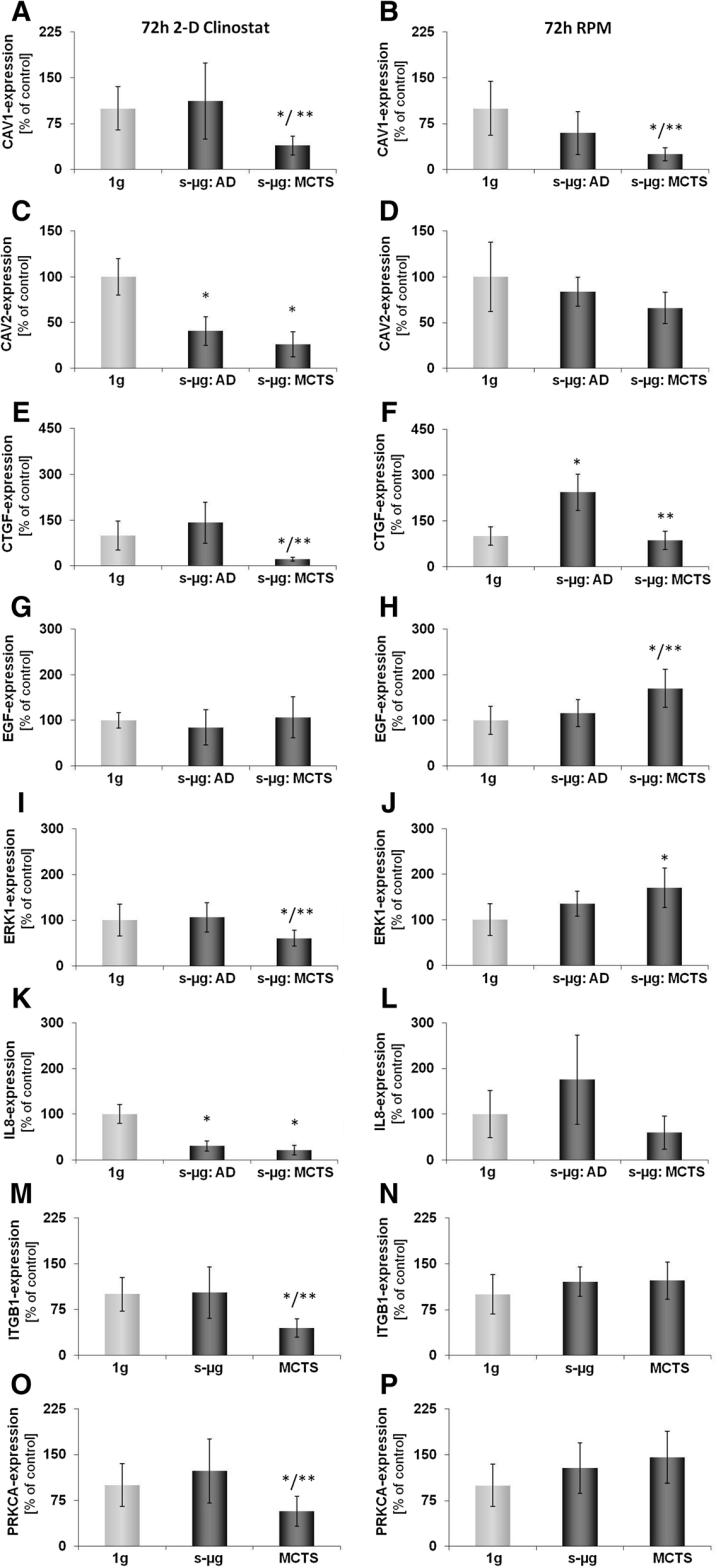
**Quantitative real-time PCR for the determination of alterations in gene-expression of selected genes after 72 h.** CAV1 **(A, B)**, CAV2 **(C, D)**, CTGF **(E, F)**, EGF **(G, H)**, ERK1 **(I, J)**, IL-8 **(K, L)**, ITGB1 **(M, N)**, and PRKCA **(O, P)** gene expression was analyzed after 72 h exposure of the cells to 2-D Clinostat **(A, C, E, G, I, K, M, O)** or random positioning machine (RPM; **B**, **D**, **F**, **H**, **J**, **L**, **N**, **P**). After 72 h culturing, the FTC-133 cells grew adherently (AD) or within the MCTS. On both machines CAV1 **(C, D)** was down-regulated in the MCTS cells and CTGF **(E, F)** was differently expressed in AD and MCTS, respectively. All results are shown as mean ± standard deviation (SD) of n = 10 independent samples, with significance indicated by *P < 0.05 vs. 1g, **P < 0.05 vs. s-μg: AD.

After 72 h of exposure to the CN or the RPM, cells had parted into adherent cells (AD) and cells forming multicellular tumor spheroids (MCTS), which floated in the supernatant (Figure 1 I). At this time, two fractions were harvested from the CN and the RPM, respectively. Subsequently, it was investigated how the expression of the selected genes (Table 1) had been regulated within the 4 cell samples indicated in Figure 3 as compared to the corresponding static ground controls (1 *g*). Interestingly, the regulation of *CTGF* and *CAV1* split on both machines equally. In adherent cells, *CTGF* remained up-regulated and *CAV1* unchanged, while in MCTS the expression of these two genes was decreased (Figure 3 E-F). Other genes have also changed their expression behavior, but differently, when incubated on the two machines. Exposure of FTC-133 cells to the RPM led to an up-regulation of *ERK1* and *EGF* gene-expression in MCTS, but did not affect one of the selected genes in AD cells. In contrast, exposure of the same type of cells to CN triggered down-regulation of *ERK1*, *ITGB1,* and *PRKCA* gene-expression in MCTS only (Figure 3 I, M, O), but decreased expression of *CAV2* and *IL8* in both, AD and MCTS cells (Figure 3 C, K).

The translation from mRNA to protein is a complex process, and results obtained on mRNA level do not necessarily reflect the situation on the protein level. Therefore, investigations of CN- and RPM- related effects on cytokine release were performed additionally using Multi-Analyte Profiling Technology (MAP).

### Cytokine release of FTC-133 cells after 72 h exposure to RPM and CN

Concentrations of selected cytokines within the various culture supernatants were determined by MAP in order to estimate the secretion activities of the cells (Table 2). As compared to the relevant 1*g* ground controls, significantly higher amounts of GM-CSF, IL-6, IL-8, BDNF, Eotaxin-1, ICAM1, IL-1α, IL-1β, IL-1Ra, IL-12p40, IL-15, IL-17, IL-23, MMP-3 and SCF were detected in the supernatants of FTC-133 cells cultured on the RPM for 72 h. In contrast, significantly lower quantities of GM-CSF, IL-6, IL-8, MIP-1α, MIP-1β and BDNF were detected in cell supernatants of CN samples than in 1*g* control supernatants (Table 2) after 72 h cell culturing. After cultivation on both machines, however, a tendency of the cells to increase eotaxin-1 and to decrease VEGF secretion was observed in a comparable manner (Table 2).

**Table 2:** Cytokines detected in supernatants of FTC-133 cells after 72 h incubation

	**2D Clinostat**			**Random positioning machine**		
**Factor**	**LDD (pg/mL)**	**72 h 1g (pg/mL)**	**72 h s-μg (pg/mL)**	**LDD (pg/mL)**	**72 h 1g (pg/mL)**	**72 h s-μg (pg/mL)**
**GM-CSF**	3.2	1536 ± 166	1172 ± 31*	9.3	46.8 ± 6.9	110.8 ± 12.0*
**IFN-γ**	0.49	n.d.	n.d.	0.58	n.d.	n.d.
**IL-2**	1.0	1.9 ± 0.33	n.d.	2.6	n.d.	n.d.
**IL-3**	1.1	n.d.	n.d.	2.0	n.d.	n.d.
**IL-4**	4.4	n.d.	n.d.	3.2	n.d.	n.d.
**IL-5**	1.0	n.d.	n.d.	0.73	n.d.	n.d.
**IL-6**	0.84	282 ± 37	204 ± 17*	0.85	43.8 ± 8.4	70 ± 15.6*
**IL-7**	2.1	n.d.	2.1 ± 0.3	5.5	n.d.	n.d.
**IL-8**	0.63	7604 ± 410	5586 ± 267*	0.49	226 ± 31	350 ± 36*
**IL-10**	1.1	n.d.	n.d.	1.5	n.d.	n.d.
**IL-18**	3.7	n.d.	n.d.	3.2	n.d.	n.d.
**MIP-1α**	8.2	3086 ± 390	2198 ± 379*	6.0	113 ± 18	137 ± 24
**MIP-1β**	4.0	261 ± 46	166 ± 28*	4.6	n.d.	n.d.
**MCP-1**	4.6	47 ± 6	40 ± 3	4.9	n.d.	n.d.
**TNF-α**	2.9	8.9 ± 1.2	7.0 ± 1.3	3.1	n.d.	n.d.
**TNF-β**	2.3	2.3 ± 0.4	n.d.	4.4	n.d.	n.d.
**BDNF**	3.0	20 ± 1.7	16 ± 1.7*	6.2	41.2 ± 4.6	76.6 ± 4.7*
**Eotaxin-1**	3.7	11.8 ± 1.8	12.4 ± 2.3	13	75.4 ± 9.9	99.4 ± 9.8*
**ICAM1**	270	360 ± 74	350 ± 73	620	1328 ± 274	1840 ± 150*
**IL-1α**	0.24	n.d.	n.d.	0.19	0.85 ± 0.11	1.60 ± 0.29*
**IL-1β**	0.3	n.d.	n.d.	0.25	1.14 ± 0.22	2.02 ± 0.34*
**IL-1ra**	8.7	n.d.	n.d.	14	127 ± 38	226 ± 24*
**IL-12p40**	16	n.d.	n.d.	17	128 ± 21	206 ± 29*
**IL-12p70**	4.8	7.8 ± 0.6	7.3 ± 0.9	4.5	36.0 ± 4.2	46.2 ± 6.1
**IL-15**	39.0	n.d.	n.d.	41	142 ± 27	208 ± 28*
**IL-17**	0.51	1.14 ± 0.32	1.0 ± 0.16	0.6	2.5 ± 0.4	4.5 ± 0.6*
**IL-23**	51	n.d.	n.d.	69	590 ± 117	868 ± 78*
**MMP-3**	5.8	260 ± 22	256 ± 19	7.4	51.2 ± 11.3	90.8 ± 11.1*
**SCF**	13	24 ± 4	19 ± 2	9.0	59.0 ± 16.1	94.6 ± 11.5*
**VEGF**	1.4	3062 ± 539	2814 ± 309	4.2	6048 ± 791	5044 ± 677

Values are given with mean ± SD; 1*g*, corresponding ground control; n.d., not detectable; *P <0,05 for device sample vs. corresponding 1*g* ground control; LDD (Least Detectable Dose)-determined as the mean ± 3 standard deviations of 20 blank readings.

## Discussion

### Spheroid formation occurs on both machines

The main result of this study is that FTC-133 cells form spheroids on the CN as well as on the RPM like they did during the Shenzhou-8 spaceflight in real microgravity [1,2]. During the MCTS formation process, the cells were separated into two populations. One continued adherent cell growth, while the other one detached and formed MCTS. Cultivation on the CN and the RPM resulted in spheroid formation like it was observed in real microgravity. Hence, both machines equally influence the cells in this respect. However, different device-specific alterations of gene-expression and cytokine secretion were found after 4 and 72 h cultivation, indicating device-specific characteristics. Common effects could be due to the prevention of sedimentation. Nevertheless, data from real microgravity are needed to clarify the situation and to control for any possibly confounding variables in the GBF and to choose the appropriate microgravity approach. A review on the suitability of diverse ground-based facilities was given by Herranz *et al.,* indicating that both, CN and RPM are generally suitable for adherent mammalian cells [6]. Nevertheless, it is also clearly stated, that although some similar results in s-μ*g* and real μ*g* were achieved, extensive studies have to be performed for each biological system and thus, individually for each type of the cell culture of interest [6]. For future studies, equal time frames, hardware and procedures should be used in space and ground-based studies in order to exclude an impact of external factors influencing the results.

Spheroids formed scaffold-free under the condition of microgravity are valuable models for tumor research [1,3,11], as they resemble the *in vivo* situation much better than 2D monolayer cultures or spheroids grown on Earth using liquid overlay or spinner flask techniques [12,13], which consist mainly of concentric layers surrounding central necrotic cells by a thin shell [14]. The importance of studies on tumor cells exposed to microgravity was recently reviewed by Becker and Souza [15]. However, MCTS generated under simulated microgravity conditions show viable cells throughout the whole body and lack necrotic centers, even if the thyroid cancer cells (ML-1) had formed spheroids with diameters of up to 300 μm [3]. The similarity between *in vivo* tumors and RPM- or CN-derived MCTS is explained by the following hypothesis: On the RPM or the CN, cells can undergo a transition from a 2- to a 3-dimensional growth, with the adherent layer serving as a starting point. Therefore, cell-cell contacts are established by forces of biochemical components expressed on the cell surfaces in a cell type specific manner under low shear forces [3]. Results of former RPM experiments with the two human follicular thyroid carcinoma cell lines FTC-133 and CGTH W-1 showed differences in the size of spheroids formed which were correlated to their capability to bind to fibronectin [4].

### Genes and proteins playing a possible role in spheroid formation

As compared to the corresponding 1*g* controls, higher amounts of the selected cytokines were frequently detected in supernatants obtained from RPM cultures and lower quantities in those harvested from CN cultures, respectively (Table 2), clearly indicating device-specific differences. Interestingly, VEGF secretion indicated a tendency of down-regulation, while eotaxin-1 suggested up-regulation on the CN as well as on the RPM after 72 h (Table 2). Former investigations of culture supernatants of samples cultured on the RPM or returned from the Shenzhou-8 spaceflight experiment showed a different picture. No changes of EGF and VEGF secretion were observed after real microgravity exposure, while both cytokines were significantly down-regulated after a 10d-RPM-exposure [2]. The different cytokine concentrations could be due to a change of the secretion activity during prolonged incubation under microgravity when the transition from a 2- to a 3-dimensional growth is completed as it has been shown for human endothelial cells [16,17]. A comparison of cytokines in the supernatant appears only possible during incubation within a few days, as investigation of IL-6 after 1 and 3 days performed in this and a former study [10] suggested. VEGF prevents apoptosis in thyroid carcinomas in an autocrine manner [18]. Its reduction may contribute to an enhanced apoptotic rate in thyroid cells cultured on the RPM [3]. Eotaxin-1 induces changes in the cytoskeleton and cell morphology and thus could favor the transition from a 2- to a 3-dimensional kind of growth [19].

Although spheroids were not seen in the cultures on both machines after 4 h, a change in gene expression activity was already expected based on earlier parabolic flight experiments [20-22]. Indeed, three of the selected genes were found to be changed. Most interesting of them is *CTGF’s* significantly enhanced expression as compared to the corresponding 1*g* controls. It remained up-regulated for another 68 h in AD cells on both machines, while the MCTS cells showed *CTGF* mRNA concentrations even lower than the control cells after 72 h of culturing. The split of *CTGF* gene expression between adherent and MCTS cells corresponds nicely to earlier data from 10 d RPM and spaceflight experiments, which indicated more mRNA in AD than in MCTS cells [2] and could therefore be a microgravity-dependent process involved in spheroid formation. *CTGF* was found to be over-expressed in papillary thyroid carcinoma correlating with metastasis, size and clinical stage [23]. It was also suggested to play an important role in angiogenesis and tumorigenesis of prostate cancer [24]. Its reduction in MCTS cells could hint to a diminished aggressiveness of cancer cells incorporated in MCTS (manuscript in preparation).

Besides *CTGF*, genes of adherent cells remained unaffected until 72 h on the RPM, while expression of *CAV1, EGF* and *ERK1* had been altered in the MCTS. In contrast, after 72 h on the CN, *CAV2* and *IL8* expression were changed in AD cells and *CAV1*, *CAV2*, *CTGF*, *ERK1* and *IL8* in the MCTS. Most interesting of these observations was a down-regulation of *CAV1* and an up-regulation of *ERK1* in MCTS cells after three days following an at least one-day-lasting stability of these genes [10,25], which differ from the *CAV2* gene that is stable only for 4 h (Figure 2) but down-regulated after 24 h and 72 h [10]. Caveolin-1 is an integral membrane protein and plays crucial roles in the regulation of cellular proliferation, differentiation and apoptosis [26]. The down-regulation of caveolin-1 appears to enhance the capacity of the cell to incorporate in a tissue [27]. Furthermore, the expression of this protein seems to be gravisensitive, because an overexpression in healthy mice staying in space had been observed [28]. Since *CAV1* is an obviously gravisensitive gene and its product influences the incorporation of cells in tissue, we conclude from our observations that *CAV1* may play a role in microgravity-dependent MCTS formation.

### Device-dependent cell modification

Unexpectedly, most of the selected genes and cytokines were differently expressed or secreted, respectively, depending on which kind of simulation device MCTS were formed. We conclude that during MCTS formation considerable alterations of various cellular molecules occur. Some of them might not be directly related to the process of 3D cell aggregation, but by products generated by device-dependent modifications [29]. A comparative study in space will help to discriminate microgravity-induced alterations from other physical stimuli due to vibration, shearing forces, etc. generated by the simulators. The 2D clinostat rotating constantly at 60 rpm generates residual accelerations below 0.012 *g* within the distance of ±3 mm around the center of the flask [30]. Cells located at further distance from the rotation axis are exposed to accelerations reaching up to 0.036 *g* at about ±9 mm. Significant differences in gene expression within these acceleration intervals have been shown by Eiermann *et al.*[30]. Based on these findings, only cells within the central 6 mm (≤0.012 *g*) of a slide were harvested and analyzed in the present study. An exception had to be made for supernatants which were collected in total, as a distinction between different acceleration levels was not possible. Therefore, supernatants were enriched by cytokines of all cells from the flasks. Whether gravity forces in a range of 0.012 *g* and 0.036 *g* have a separated influence on the cell behavior has not yet been clarified [30,31]. Either coating of the flasks in the parts outside the area of interest in order to prevent initial cell attachment or a different geometry of the flasks themselves, with an enlarged area along the rotation axis would be convenient to avoid a mixture in cytokine release.

Concerning clinorotation, no relative fluid motion is assumed in a closed completely filled and air bubble-free container exposed to the CN due to its linear and constant acceleration mode [32]. In contrast, chaotic fluid motion is caused on a RPM operating in real random mode due to random changes of speed and direction of the platform movement [33-35]. Although the magnitude of forces of fluid motion, which does not exceed 0,44 dyn/cm^2^[35], is below that required to trigger cell detachment from surfaces [36,37], it is assumed to be high enough to induce an enhanced convective fluid mixing, which may support nutrient supply and removal of metabolic products and thus optimize the culture conditions for a cell.

Residual accelerations for RPM exposure have to be considered for all 3 *g*-vector components that means in x, y and z directions. Exemplary measurements of a RPM running at 60–120 °/s showed accelerations due to gravity of -1 to +1 *g* for each direction [38]. A slower rotation speed produces less residual acceleration but requires a longer time for averaging of the g-vector. The relation of rotation speed, resulting accelerations and the time a systems needs to detect the alterations of the influence of gravity has to be considered. Even though less residual accelerations can be achieved through a decreased rotation speed, a longer exposure stimulus will be inevitably connected which can possibly lead to a permanent stress stimulus, if sensed by the cells [39].

Shear stress has to be considered as a variable differing between the devices. As shown by Goodwin *et al.* BHK-21 cells exposed to 0.51 dyn/cm^2^ consumed 60-70% less glucose, but achieved higher cell counts and aggregation than cells exposed to 0.92 dyn/cm^2^, both on the integrated rotating wall vessel (RWV) [40]. Furthermore, experiments by Hammond *et al*. on primary cultures of human renal cortical cells exposed for 6 days to either real microgravity on a space shuttle or cultured on ground-based devices as controls demonstrated 1632 gene changes after the space flight, 5 gene changes after centrifugation and 914 gene changes after cell culturing on the RWV, if a threshold of more than ± three-fold change was set. In addition, as many gene changes in reciprocal as in the same direction were observed, when results from RWV and space cultures were compared [41]. The balancing forces, such as shear, used to offset gravity in RWV systems were suggested to be responsible for changing the character of the culture [41]. Further work by Kaysen *et al*. supports this hypothesis, showing a shear stress dependence of selected *de novo g*ene and protein expression during renal epithelial cell culture in RWV [42]. Therefore, different shear stress situations on both devices used in our study seem to be a cause of differences in the results obtained. The problem of internal shear forces due to residual accelerations has been recognized not only in ground-based facilities, but was also predicted to be responsible for differences between 1 g ground controls and 1 *g*-in-flight controls using a 1 *g*-reference centrifuge in space, as described by van Loon *et al.*[43]. An adjustment of the culture flasks to the direction of the forces is suggested in order to obtain an optimal outcome and comparability of the data. Further experiments are required to clarify stress and gravity-related effects and to determine threshold levels for response time and optimal rotation speed in order to simulate optimal microgravity conditions on ground.

Also vibrations are often suggested as a critical parameter in microgravity simulation. As the corresponding 1 *g* controls were cultured next to the devices in the same incubator, the vibration effect should be neglected as both samples 1 *g* and s-μ*g* are exposed to the same external stimulus. The same matter has to be addressed in parabolic flights especially in combination with periods of hypergravity. Experiments with respect to vibration and hypergravity have been performed on two human follicular thyroid cancer cell lines (ML-1 and CGTH W-1) and endothelial cells (EA.hy 926), all suggesting that microgravity effects are stronger than the opposing vibration and hypergravity effects [20-22]. Nevertheless, additional vibration experiments for FTC-133 are of interest as final validation of ground- based and future space data.

A further aspect possibly influencing the results are the different culture containers used due to the geometry of the devices. As the same flasks were used for s-μ*g*-and corresponsing 1 *g*-samples and results were analyzed relatively to the in parallel obtained 1*g*-data, an influence should not be expected for the PCR data. Concerning the MAP data, a possible effect cannot be excluded due to different volume and area proportions.

For the CN, special slide flasks had to be used in order to utilize the harvesting area on the rotation axis to a maximum extent and to adjust a larger amount of samples on the machine. This leads to differences in the proportion of cells/cm^2^ and cells/ml for both devices which might have an impact on the cytokine measurements in the supernatant and might influence interaction and signaling of the cells. For the clinostat, a maximum of 0.056 × 10^6^ cells/cm^2^ were used for 4 h-experiments. Longer experiments of 24–72 hours were seeded with less density in order to prevent overgrowing in the limited culture area of only 9 cm^2^. A culture volume of 20 ml resembles 0.025 × 10^6^ cells/ml. For the RPM equal amounts of cells were used for all time frames, as a bigger culture area of 75 cm^2^ and a larger volume of 320 ml culture media was availabe. This leads to 0.053 × 10^6^ cells/cm^2^ and 0.0125 ×10^6^ cells/ml. As a consequence, differences in autocrine and paracrine signaling between the cells, due to the differences in cell to area/volume proportion as well as different fluid behavior due to the geometry of the culture flask, clinorotation and random rotation have to be taken into consideration. The hypothesis, that *in vitro* responses of cells to modified inertial environments might be a manifestation of modified extracellular convective flow has been suggested by Paul Todd already in 1992 [44]. For future experiments, equal culture conditions should be used in order to obtain an optimal comparability of the data. Still, the unique conditions of real microgravity with the loss of gravity-dependent convection and negligible hydrodynamic shear have to be considered when comparing real and s-μ*g* results [15,44,45].

## Conclusion

On both, the CN and RPM, spheroid formation of human thyroid cancer cells was observed, whereas, besides a few exceptions, a considerable number of selected genes or cytokines were expressed or secreted differently, although equal kinds of cells formed MCTS on the two machines. The exceptions were *CAV1* and *CTGF* genes as well as VEGF and eotaxin-1 cytokines. We consider them involved in the process of spheroid formation, because their changes consistently accompanied the MCTS formation in similar manner, when cell sedimentation is prevented by RPM or CN or even in real microgravity in space. The study shows the advantage of searching for gravity-sensitive genes and proteins in comparative approach using different machines for microgravity simulation. Our study clearly shows the necessity to verify results from ground-based simulation approaches to the ones obtained in real microgravity conditions to avoid misinterpretations, to learn and to understand device-specific characteristics and finally choose the appropriate simulation approach.

## Methods

### Culturing of FTC-133 cells

The human follicular thyroid carcinoma cell line FTC-133 [46] was cultured in RPMI-1640 medium at 37°C and 5% CO_2_. The medium was supplemented with 100 μg/mL streptomycin, 100 U/mL penicillin and 10% FCS (all Biochrom, Berlin, Germany). One day prior to the CN experiments, cells were seeded in 9 cm^2^ slideflasks (Thermo Scientific, Roskilde, Denmark). A cell count of 5 × 10^5^ cells was disseminated for 4 h experiments, 4 × 10^5^ for 24 h and 2 × 10^5^ for 72 h experiments. For the RPM experiments, cells were grown in T75 cell culture flasks (Sarstedt, Numbrecht, Germany). Cells were seeded at a density of 4 × 10^6^ cells per flask. The cells were randomized to be cultivated as static ground controls (1 *g*) or under simulated microgravity conditions (s-μ*g*) on either a RPM or a CN. Ground controls were always placed next to the device in the same incubator. Cells and supernatants were harvested after 4 h or 72 h on ice. The supernatants were aspirated and centrifuged at 4°C. Afterwards, the fluid was transferred to another tube and frozen. The pellet was fixed with RNAlater. After removal of the culture supernatant, cells which remained adherent during incubation, were washed once with PBS and then fixed with RNAlater. For this, in case of the CN the slides were remove from their flask and were slowly dipped first in PBS (5 s) and then transfered to RNAlater until harvesting. T75 culture flasks from the RPM were slowly filled with 10 ml PBS while standing vertically, and were very carefully brought into horizontal position to avoid any disturbance of the cells. PBS was aspirated again before the addition of RNAlater. Afterwards, the cells cultured on the RPM were scraped off the whole bottom surface, while cells cultured on the CN samples were harvested from the inner 6 mm of the slide flask only, because cells of this part experience accelerations of ≤0.012 *g*[30].

### Random Positioning Machine (RPM) and 2D clinostat (CN)

For a comparative methodical approach, cells were either cultivated on the Desktop RPM manufactured by Dutch Space (an EADS Astrium company, Leyden, Netherlands) [8] or the Fast Rotating 2D clinostat (DLR, Cologne, Germany) [30]. The Desktop RPM was operated in real random direction mode (60 °/s) and equipped with four T75 flasks per run, fixed to the ground plate, giving a maximum distance of 7.5 cm from the center of rotation.

The 2D clinostat constantly rotated with 60 rpm and was loaded with four slideflasks on each of the 6 parallel rotating axes, summing up to a total of 24 flasks per run. Both devices were placed inside an individual incubator with temperature of 37°C and 5% CO_2_. Prior to the experiment, all flasks were completely filled with media avoiding air bubbles carefully, in order to reassure a minimization of turbulences. For harvesting of CN samples, cells of 16 flasks were pooled. Ten samples (n = 10) were collected for each condition: CN, CN corresponding 1*g* control, RPM and RPM corresponding 1*g* control.

### Phase contrast microscopy

Phase contrast microscopy was performed for visual observation of the morphology of the cells, using the Axiovert 25 Microscope (Carl Zeiss Microscopy, LLC, United States).

### Cytokine measurements by Multi-Analyte Profiling technology

The release of cytokines was investigated via Multi-Analyte Profiling (MAP) as previously described [16,47,48]. For each condition, five supernatants were collected after 72 h and stored at -80°C until testing. The MAP was carried out by Myriad RBM (Austin, Texas, USA) using the Human Cytokine MAP A and B. Briefly, Micro beads carrying specific antibodies, directed against the target analytes, are used to detect the cytokines. A second biotinylated reporter antibody is added, followed by an excess of streptavidin-phycoerythrin solution to develop the multiplexes and thereby enabling the quantification of the cytokines released, via fluorescence detection [49].

### RNA isolation and quantitative real-time PCR

RNA isolation and quantitative real-time PCR were performed according to routine protocols [25,50,51]. For CN samples, only the inner 6 mm were harvested with a scratching device. RPM samples were harvested in total. RNA was isolated using RNeasy Mini Kit (Qiagen, Hilden, Germany) following manufacturer instructions. DNase (Qiagen, Hilden, Germany) was added in the process of RNA isolation, in order to diminish residual DNA contaminations. The RNA was quantified via Photometer Ultrospec2010 (Amersham Biosciences, Freiburg, Germany). Reverse transcription was performed using the first strand cDNA synthesis kit (ThermoFisher Scientific, Waltham, US), following manufacturer’s instructions. Quantitative real-time PCR was utilized to determine the expression levels of target genes, shown in Table 1, using the 7500 Real-Time PCR System (Applied Biosystems, Darmstadt, Germany). cDNA-selective Primers were designed to span exon-exon boundaries and to have a T_m_ of ∼ 60°C using Primer Express software (Applied Biosystems, Darmstadt, Germany), and were synthesized by TIB Molbiol (Berlin, Germany). All samples were measured in triplicate and normalized to the housekeeper 18S rRNA. Comparative C_T_ (∆∆C_T_) methods were used for relative quantification of transcription levels, with 1 *g* set as 100%.

### Statistical Evaluation

Statistical Evaluation was performed using SPSS 15.0 (SPSS, Inc., Chicago, IL, USA). The Mann–Whitney-U-Test was used to compare 1*g* and s-μ*g* conditions, as well as s-μ*g* adherent cells and s-μ*g* MCTS cells. All data is presented as mean ± standard deviation (SD) with a significance level of p < 0.05. * indicating the comparison of 1*g* vs. s-μ*g* and ** representing the comparison of s-μ*g* AD vs. s-μ*g* MCTS.

## Abbreviations

BDNF: Brain-derived neutrophic factor; CAV: Caveolin; CN: Clinostat; CTGF: Connective tissue growth factor; EGF: Epidermal growth factor; ERK: Extracellular signal-regulated kinase; FCS: Fetal calf serum; FTC: Follicular thyroid cancer; GBF: Ground-based facilities; GM-CSF: Granulocyte-macrophage colony-stimulating factor; IFN: Interferon; IL: Interleukin; ITBG1: Integrin beta 1; LDD: Least detectable dose; MAP: Multi-Analyte Profiling; MCP: Monocyte chemotactic protein; MCTS: Multicellular tumor spheroids; MEK: Mitogen-activated protein kinases; MIP: Macrophage inflammatory protein; MMP: Matrix metalloproteinase; MRNA: Messenger ribonucleic acid; PCR: Polymerase chain reaction; PDGFR: Platelet-derived growth factor receptor; PRKCA: Protein kinase C alpha; RBM: Rules-based medicine; RPM: Random positioning machine; RWV: Rotating Wall Vessel; SCF: Stem cell factor; SD: Standard deviation.

## Competing interests

The authors declare that they have no competing interests.

## Authors’ contributions

JB, DG and RH designed the study. EW and XM conducted the experiments. EW and JB drafted the manuscript. JP, MW and MI supervised and supported the ground-based experiments in Magdeburg. DG supervised the ground-based experiments in Aarhus and helped with the manuscript. RH and MB supervised and supported the ground-based experiments in Cologne. MG was responsible for the design of the 2D clinostat. All authors read and approved the final manuscript.
